# Pharmaceutical Terminology in Ancient and Medieval Time – *andrachne, chrysocolla* and Others*

**DOI:** 10.18778/2084-140X.13.49

**Published:** 2023-12-30

**Authors:** Barbara Zipser, Andrew C. Scott, Robert Allkin, Peretz Gan, Andreas Lardos, Rebecca Lazarou, Efraim Lev, Mark Nesbitt, Kristina Patmore

**Affiliations:** Royal Holloway, University of London, Egham Hill, Egham, Surrey, TW20 0EX, United Kingdom; Royal Holloway, University of London, Egham Hill, Egham, Surrey, TW20 0EX, United Kingdom; Royal Botanic Gardens Kew, Kew, Richmond, London TW9 3AE, Great Britain; Al Alim Medicinal Herb Center, Moshav Zippori 49 Hamovil 1791000, Israel; Zürcher Hochschule für Angewandte Wissenschaften, Gertrudstrasse 15, 8400 Winterthur, Switzerland; Royal Botanic Gardens Kew, Kew, Richmond, London TW9 3AE, Great Britain; University of Haifa, 199 Aba Khoushy Ave. Mount Carmel, Haifa, Israel; Royal Botanic Gardens Kew, Kew, Richmond, London TW9 3AE, Great Britain; Royal Botanic Gardens Kew, Kew, Richmond, London TW9 3AE, Great Britain

**Keywords:** pharmacognosy, Byzantine medicine, medicinal plants, medicinal minerals, medical history, Greek medicine, *materia medica*

## Abstract

Ancient and medieval pharmacological and medical texts contain a substantial amount of plant and mineral names. In some cases, the identification is straightforward. But for the majority of the data, we are unable to identify these ingredients with high certainty. In this paper, we discuss a selection of plant and mineral names both from a humanities and sciences point of view. In one case, the scientists were even able to examine a plant in situ. The conclusion of our paper is that a close collaboration between sciences and humanities is essential to avoid mistakes in the identification of *materia medica*.

## Introduction

Ancient and medieval medical authors mention a significant number of plants and minerals that were meant to be used for the preparation of pharmaceuticals. In some cases, we can be confident that we know what these sources describe. Olive oil, or olives specifically, would be one such product. We have an abundance of information on olives throughout the centuries, from a broad selection of genres, there is a continuity in olive cultivation and use, and olives still have a similar name in many languages^1^. What is more, a recent genetic study concluded that the olive trees that can be found today throughout the Mediterranean go back to a cultivar from Turkey or Syria that was grown 6000–8000 years ago^2^. This also gives us a strong indicator to what extent olives may or may not have changed their properties over the centuries.

However, for the vast majority of plants mentioned in ancient or medieval sources we cannot be certain about the identity of the plant cited. Often, it is impossible to determine what a plant name in an ancient or medieval text would equate to in our modern world.

It would be easy to blame the underlying scientific system of Classical antiquity for this, which may in some aspects be out of date^3^. While these differences in the perception and definition of plants and minerals certainly play a part, our own use of terminology may also be inaccurate. We may use synonyms or homonyms, dialectal words, or inaccurate botanical nomenclature. For instance, the term “marigold” is associated with at least 18 different species of plant^4^, including species from different genera such as *Calendula* L., *Tagetes* L. and *Bidens* L. which have little in common other than they share the same colloquial name. Such names are part of ordinary language, their use varying between regions, or even between neighbouring villages, and their use changes over time. Scholars who do not have a background in botany often also use these colloquial terms in their publications.

In contrast, scientific plant names are controlled. These names must be formally published in accordance with the International Code of Nomenclature for Algae, Fungi, and Plants^5^, including providing a description and citing one or more voucher specimens that may be referred to in perpetuity.

Yet even this system does not necessarily allow us to identify a given plant, not even if we are able to view and examine a specimen. We encountered such a situation during a recent research trip to Haifa, when Peretz Gan, the owner of Al Alim Medicinal Herb Center and himself a knowledgeable herbalist, asked the group whether they could determine the identity of a tree in his orchard. Our delegation included a number of capacities in the field, who examined the plant, but could not come to a conclusion.

The shrub was originally believed to be *Commiphora gileadensis* (L.), which is in popular culture often equated with the balm of Gilead^6^, a plant gifted to King Solomon by the Queen of Sheba, as described in the book of Kings I^7^. A number of other Hebrew, Latin and Greek writings also mention plants that were believed to be the balm of Gilead^8^.

The defining part of this tradition, however, is the story about the Queen of Sheba. Here, the textual evidence is far from unambiguous. The original of this story was written in Hebrew, probably between the 6^th^ and 7^th^ century BC, and then translated into Greek, and into Latin. The Hebrew original^9^ Rg I 10, 10 uses the word וּבְשָׂמִ֛ים which goes back to the root BŠM, which can denote a number of aromatic spices. The standard lexicon in the field identifies it with “cinnamon, calamus odoratus, balsam plant” and also lists ample evidence for a more general translation of spice^10^. In the Septuagint^11^, the Greek translation of the Hebrew original, probably dating to the 3^rd^ and 2^nd^ century BC, Rg III 10, 10 the plant in question is referred to as ἡδύσματα, which is a rather vague term that can denote spices or seasonings^12^. In Vulgate III Rg 10,10^13^, the Latin translation of the Hebrew original, dating to the 4^th^ century AD, the word *aromata* is used, which merely denotes spices^14^.

This story is repeated in Josephus *Jewish Antiquities* VIII 174^15^ of the first century AD, where the plant is called ὀποβάλσαμον (*opobalsamon*) or ἀποβάλσαμον (*apobalsamon*)^16^. This plant name, *opobalsamum*, is indeed known to us from various other Greek medical sources, and here in particular Dioscorides I 19^17^, the author of a highly important treatise on pharmaceutical ingredients of the 1^st^ century AD, who also describes its medical uses.

Even this brief survey of the key evidence is enough to cause significant doubts. In tenth century BC, according to a passage of the *Old Testament* that was probably written some 300 years later, the Queen of Sheba brought a “spice” to Israel. Some one thousand years later this spice was identified with a plant that grew in these locations by Dioscorides, of which we then have a detailed description.

Therefore, the only matter we can conclude with relative certainty is that a plant described in Dioscorides existed during his life time, in the first century AD.

But whether this plant is the same as the ones grown in the Near East today is an entirely different question – and whether all the plants that are believed to be *Commiphora gileadensis* (L.) C.Chr. are actually one and the same species. A confusion like this might seem unlikely, given that these plants were supposedly either native to this area or had been grown there for millennia. But it might happen easier than one might assume at first sight: the trees at Al Alim were grown from seeds that were provided as *Commiphora gileadensis* by a trusted source, and they have been cultivated for some ten years. However, recently it has been suggested that these trees could indeed be *Bursera fagaroides* (Kunth) Engl. – a new world plant that looks very similar or even the same to the naked eye. Could there have been a mix-up somewhere down the line? And to what extent can we actually be certain that the trees labelled as balm of Gilead or *Commiphora gileadensis* growing in Israel today, or elsewhere, are even one and the same species? What is certain, though, is that some of plants believed to be *Commiphora gileadensis* L. do indeed have pharmaceutical value^18^.

In the following, we are going to discuss two more samples, a plant and a mineral, that would seem easy to identify from a modern lay perspective. But precisely this can be treacherous.

## Andrachne and andrachle

*Andrachne*, or ἀνδράχνε in the original Greek, is frequently mentioned in Greek medical texts^19^. Anecdotally, a lot of scholars working in the field would instinctively say that it is *Portulaca oleracea* L. The standard dictionary for Classical Greek states that it is the Attic form of ἀνδράχλε (*andrakhle*), which it equates with *Arbutus andrachne*^20^. Yet Lily Beck’s Dioscorides translation, which many use as a dictionary of plant names, confidently identifies *andrachne* with *Portulaca oleracea* L.^21^ This leaves us already with two very different plants, a tree and a shrub from two entirely different plant families.

Kriaras, the standard dictionary of medieval Greek, translates *andrachne* with two other Greek terms, αντράκλα (*antrakla*) and γλιστρίδα or γλυστίδα (*glistrida*) without a further attempt at identification, other than stating that it is a type of plant^22^.

But what does the primary source evidence say? Let us start from scratch. The first comprehensive and authoritative work on ancient botany, which dates to the early 3^rd^ century BC Greece, Theophrastus *Historia Plantarum* I 9, 3 and IV 15, 1, mention a tree called *andrachle*^23^. Pliny the Elder, a Roman author of the 1^st^ century AD, on the other hand provides this curious account in XIII 40, 120 of his *Naturalis Historia*^24^:

Andrachlen omnes fere Graeci porcillaceae nomine interpretantur, cum sic herba et andrachne vocetur unius litterae diversitate. Cetero andrachle est silvestris arbor neque in planis nascens, similis unedoni folio tantum minore et numquam decidente cortice non scabro quidem sed qui circumgelatus videri possit tam tristis adspectus est.

*Andrachlen* almost all Greeks translate with the name *porcillacea*, whereas this plant is also called *andrachne* with a difference of one letter. Furthermore, *andrachle* is a forest tree and it does not grow on plains, it is similar to *unedo* just with a smaller and never shed leaf, and a bark that is not rough in a way but which could appear to be frozen around it, this is how sad its appearance is.

Here, we can see a curious phenomenon. Pliny quotes Greek terminology, in one case an inflected form, but he writes it in Latin letters. At this point of time, a Greek book would most likely have been written in a handwriting in which the letter L and N look very similar – Λ and N, with just one line difference, which could suggest a scribal error. However, as we already know that both forms, with L and with N are well attested in other sources, which are unrelated we can assume that this was not the case, and that this palaeographical feature is just a mere coincidence.

Now that a transmission error can be excluded, what does Pliny say? According to him, the most common form of the word is *andrachle*^25^, whereas *andrachne* can also be found. He equates it, based on his sources, with *porcillacea*, a herb. A lesser known use of the word *andrachle* would be a term for a tree, which he describes in detail^26^. All we can take away from his testimony is that at his time in the first century AD, there were two plants associated with one name, and one with the other.

Dioscorides II 124 mentions *andrachne*, but does not describe what it looks like. He seems to assume that everybody would be familiar with this plant^27^. All we can take away from this passage is that the name was spelled with N.

Two later sources provide more information. A pseudo Galenic work, *Lexicon Botanicum*, describes it as follows^28^:

ἀνδράχνη τὸ χοιροβότανον ἤγουν ἡ ἀντράκλα.

*Andrachne* is *choirobotanon* that is *antrakla*.

In this instance, the lexicon equates the word forms spelled with L and N and adds another term. This word suggests that it is a herb rather than a tree.

A similar text says^29^:

ἀνδράχνη ἡ γλυστίδα

*Andrachne* is *glystrida*.

Oribasius, a prominent medical writer of the 4^th^ century AD, mentions *andrachne* very frequently. In one passage, he compares another plant to “garden *andrachne*”. This suggests that there was a domesticated and a wild form of the plant^30^.

This is also supported by another source, *Hippiatrica Berolinensia* VII 9, 5, a Greek veterinary medical compilation with a complex history that makes it difficult to date^31^:

ἀνδράχνη λάχανόν ἐστιν ἄγριον κηπουρικόν.

Andrachne is a wild garden vegetable.

Simon of Genoa, a 13^th^ century AD physician working at the papal court in Rome, mentions the plant in his multilingual dictionary of medical terminology, *Clavis Sanationis*, too^32^. See for instance these corresponding entries:

*Portulaca dicitur grece andragni arabice vero bachalachancha*.

*Portulaca* is called *andragni* in Greek but in Arabic *bachalchancha*

*Andragne grece portulaca ut apud Dy. sed grecus dicit andrachni*…

*Andragne* is in Greek *portulaca* as in Dioscorides but the Greeks say *andrachni*…

Simon conducted in-depth research, which was based both on oral and written sources. The extent of his research is breath taking. He travelled extensively and he must have had access to a substantial number of libraries in the Mediterranean area. Simon equates *andrachne* with *portulaca* and an Arabic plant name, البقلة الحمقاء which is translated as “portulaca” in a standard modern Arabic dictionary, and the first part of the term actually translates to herb^33^. The plant bearing this name is also described in Al-Kindi, which provides further evidence that we are indeed dealing with a herb^34^.

The term *andrachne* is also mentioned in the medical work of Ioannes archiatrus, but only on two occasions in the late 13^th^ century vernacular commentary version does he provide *glistrida* as a synonym, in ω 43, 5 and 175, 12 respectively^35^:

ἀνδράχνης σπόρον ἤτοι γλιστρίδας

The seed of *andrachne*, that is *glistrida*

ἀνδράνχνην τὴν λεγομένην γλιστρίδαν

*Andrachne*, which is called *glistrida*

This brief survey of the key literature leaves us with doubts as to whether the common perception that *andrachne* is the Greek word for purslane is indeed correct. Not only does the term occur in two variations, with one letter difference, but it can also refer to two very different plants, a tree and a herb from different families.

This of course raises the question how ancient and medieval pharmacists would have known which plant to use. Oral traditions and practical training may have played a role. And in a case such as this, where the homonymous plants have a very different appearance, a competent herbalist could have recognised which one is required in a recipe based on the part of the plant that is used.

In the case of the plant name *andrachne*, a remedy prepared either with the seed of the plant or without specifying a plant part (which usually means that the whole plant or aerial part of the plant was used) would have referred to purslane (*Portulaca oleracea* L.). On the other hand, a remedy prepared with the fruits or the bark of the plant would have referred to the strawberry tree (*Arbutus unedo* L. or *A. andrachne* L.). However, often this kind of context is not taken into account in modern dictionaries.

The conclusion of this part of the article certainly is that one should be critical of any identifications that would seem obvious as this can lead to a false sense of security. Anecdotally, if one walks into a Cypriot greengrocer’s in London and asks for *glistrida* one is sold *Portulaca oleracea* L. It would then be easy to fall for a chain of conclusions such as *glistrida* equals *andrachne*, today’s *glistrida* is *Portulaca oleracea* L. and therefore *andrachne* must have been *Portulaca oleracea* L. This is particularly treacherous when it comes to potential pharmaceutical use of these plants.

In the following section, we are going to examine a case of a mineral that is mentioned in ancient sources.

## Chrysocolla

When it comes to the identification of minerals mentioned in ancient and medieval sources, the situation is quite similar to the problems described above in the section on medicinal plants. In some cases, such as native elements (gold, silver, copper and sulphur) the identification may be fairly clear. Gold for instance is extensively discussed in ancient sources, including its properties, and a wealth of gold objects have been preserved in the archeological record. Mercury certainly has very distinctive properties, and the ancient descriptions and methods of production meet our modern expectations^36^.

However, how people in general may identify a mineral is not necessarily the modern scientific method. A good example is the common name given to a gemstone in the British Crown Jewels: The Black Prince’s ruby. This is a large red gemstone and is in the general public understanding a red gemstone that is commonly identified as a ruby, which is a type of corundum. However, a recent investigation yielded^37^ that this gem is in fact a spinel – a quite different mineral. The mineral corundum is in some forms considered a gemstone. It is an aluminium oxide (Al_2_O_3_) and is very hard and has a trigonal crystal structure^38^. However, its colour is very variable from red (known as ruby) to blue (known as sapphire), green, pink, yellow brown and colourless, all also known as sapphire. The colours are caused by some elemental impurities; for instance, chromium gives a red colour and iron and titanium give a blue colour^39^. However, spinel is a magnesium aluminium oxide and has an isometric crystal structure and can be colourless, black, blue, red, violet, green, brown and pink^40^.

The method of naming minerals has had a very long and complex history, so much so that a more rigorous international approach was instigated in 1950 with formal international approval of names. Even so, this classification of using the chemical composition and mineral structure has been recently challenged with some additional properties and data of formation and origin^41^ also suggested to provide a better classification^42^.

It should not be a surprise, therefore, that the identification of minerals in ancient pharmaceutical literature is problematic and some commonly assumed identifications might have to be challenged and reconsidered. Unlike with plants, where an illustration or description may provide an important clue to identification, the colour and gross form of a mineral may not provide sufficient detail to identify mineral species. We can take three examples from Dioscorides^43^.

Dioscorides V, 89 mentions a mineral called χρυσοκόλλα (*chrysocolla*). The name is derived from the Greek χρυσός (*chrysos*) meaning gold and κόλλα (*kolla*) meaning glue and was first used by Theophrastus in Hellenistic times^44^ but revived as a formal mineral name in 1808^45^. Chrysocolla is a green or blue mineral that has a greasy or vitreous lustre. It is generally amorphous, compact and occurs as a crust or encrustation and often has grape-like aggregates. It is a hydrated copper silicate^46^.

As we shall see, there is no guarantee that the modern mineral meaning is the same as the ancient meaning. Dioscorides states that the best chrysocolla is the Armenian, being intensely green in colour, second best is the Macedonian, then the Cyprian. However, we know that chrysocolla (Plate, Fig. A) does not occur in Cyprus so what could the mineral be? There are a number of blue minerals with a similar form that occur in Cyprus but they all have a different chemical structure. For example, celadonite (Plate, Fig. B) does occur. This has a blue-green colour but has a monoclinic crystal structure but more importantly is a potassium silicate. The name is after the French célador for sea-green, its common colour^47^.

A more common example^48^ that is often used in ancient texts is a mineral commonly identified as lapis lazuli. Consequently, Beck’s standard translation of Dioscorides V, 91 identifies and translates the original word κυανός (*kyanos*) as lapis lazuli. The following text describes it as being produced in Cyprus from copper mines. This is a clear misidentification as the mineral does not occur in Cyprus^49^ or as a result of copper mining or smelting^50^. The mineral is widely identified in ancient pharmacological texts but again this identification may not be secure. In modern terms Lapis Lazuli is a rock type, rather than a single mineral species. The name was first used in 1636 by Anselmus de Boodt in *Gemmarum et Lapidum Historium*^51^ derived from the Latin *lapis*, rock, and the Persian *lazhward*, meaning blue. It is an uncommon metamorphic rock that occurs only commonly in Afghanistan. The blue mineral is mainly lazurite or more commonly a sulphur-rich variety of hauyne^52^.

Just these two examples make it clear that a different approach to the identification of minerals in ancient pharmacological tests needs specialist input and a lot of consideration from both the geological and the philological side. The mineral name *kyanos*, for instance, is very similar to the name kyanite, a mineral known today, but the Greek word κύανος (*kyanos*) just means blue.

Our final example is that of calamine, as mentioned in Beck’s translation of Dioscorides V, 74. The original Greek term is καδμεία (*kadmeia*), and Dioscorides states that it occurs in Cyprus. In addition, it is stated, according to Beck’s translation, that *calamine is formed as copper is heated in furnaces and soot settles on the walls and roof of the furnaces*. However calamine (Plate, Fig. C), as it is understood today is in fact a mineral of zinc. The name is no longer used and in its place two mineral names are common: smithsonite, that is a zinc carbonate and hemimorphite that is a zinc silicate. Although with different crystal structures these two minerals exhibit the same botryoidal form and cannot be distinguished without chemical analyses^53^. A confusion exists in that the common term calamine lotion is used for a mixture of zinc oxide and iron oxide. In all these examples we must be very cautious in the certainty of any mineral identification.

## Some possible approaches to the identification of minerals in ancient texts

It is important to realize that the use of a mineral in any medical treatment may be because of several factors, apart from the more superstitious thoughts about colour. Indeed colour may be caused by a number of different reasons from changes in crystal structure to the incorporation of minor or trace elements in the crystal structure. What is often forgotten is that it is rarely a particular mineral that is significant but the elements that make up the mineral and how any particular element may be made bioavailable for external or internal use^54^. We note that minerals are made up of two main components: cations and anions. Cations have a positive charge and are mainly metallic atoms whereas anions have a negative charge and are non-metallic atoms. So for example, the mineral calcite is a calcium carbonate mineral, with the main metallic species being calcium. Calcium is an important element for the working of the body^55^. This is also the case with copper^56^. However, copper may be connected to a range of anions that may be more or less easy to be made bioavailable to the body^57^. For example we have copper oxide (cuprite), copper carbonate (azurite) (Plate, Fig. D), malachite (Plate, Fig. E), copper sulphide (e.g. chalcocite), each of which may react differently in pharmacological recipes^58^. Equally, although the colour blue is most often associated with copper minerals and derivatives (e.g. copper sulphate or blue vitriol) there are many blue minerals lacking copper^59^.

One key piece of evidence may be firstly the common occurrence of the mineral, where one element is needed for a particular use thereby making it a valuable commodity^60^. The second issue is whether the mineral can be transformed in a particular recipe. In this regard we need to consider the reactivity of the anions. For example silicate minerals are difficult to dissolve whereas carbonate, sulphate and sulphide minerals may react with an acid, such as the acetic acid in vinegar. Together, then this may reveal clues to identity.

In relation to copper minerals, therefore, those of Cyprus are mentioned most often, indeed the name of the island is after copper^61^. The modern geology of Cyprus is particularly informative as well as the known historic exploitation of resources^62^. Whereas Cyprus is justifiably famous for its copper there is often a misunderstanding among non-geologists of how this occurs in Cyprus and indeed the wide range of copper minerals that exist^63^. These may be considered either as primary or secondary ores (sulphide ores or oxidation products such as carbonates or oxides) or else as different chemical associations (e.g. copper sulphides, copper carbonates, copper silicates etc)^64^. Each group may react in a different way to, for example acids such as with vinegar. We know that in Cyprus the main iron and copper ores are the massive sulphide deposits^65^ that have been worked for over 5,000 years. We can learn much also from an examination of ancient slags to understand the nature and uses of these deposits^66^. The main ore is iron sulphide (iron pyrite) with copper sulphide being a minor component^67^ (Plate, Fig. F). Bornite (Cu_5_FeS_4_) also occurs^68^ (Plate, Fig. G), but is much rarer.

Another issue relates to minor or trace elements that are present within a mineral and it may be that this rarer component is the one of interest. For example we now know that selenium is an important metallic element essential for some bodily functions^69^. The element rarely forms a mineral on its own. The element is found in several mineral species such as in over 100 copper minerals. It is possible that a mineral may be found to be of use by the chance occurrence of such a trace element.

Recent studies have indicated that selenium exerts a beneficial effect on coronary disease mortality, and that selenium plus garlic produces significant anti-cancer activity. Selenium is primarily taken up from the soil by plants as selenate (SeO_4_^2-^) or selenite (SeO_3_^2-^). The assimilation of selenate appears to follow the sulphate reduction pathway common to higher plants. Cereals and forage crops convert selenium into mainly selenomethionine and incorporate it into protein^70^.

This then leads to another possible approach. That is a consideration of the use of a mineral in an ancient recipe book for an identified condition and then the opposite – the use of an element for the treatment or identification of a condition in modern medicine. There has been rapid development of our understanding of the use of minerals in modern medical practice. A useful example is that of G. Borkow and J. Gabbay^71^. In this paper for example it is shown that copper has potent biocidal (including anti-fungal) properties.

Another approach is to consider both the ancient and modern use of minerals in medicines and indeed how lack of particular elements may themselves cause a range of medical conditions.

Ancient: There has been a significant rise of interest in ancient medicines in relation to modern cancer treatments^72^. Many of the approaches to potential cancer treatments have been summarised by Hajdu and these will be compared to treatments suggested in our texts and their current and potential use in modern medicines.

Modern: One aspect of ancient copper mining in Cyprus deposits^73^ is related to the concentration of toxic heavy metals in soils and also in wild and cultivated plant species. Many of these have serious health implications^74^. Spoil heaps derived from ancient copper workings have elevated concentrations of sulphur, zinc, copper and lead. Manganese concentrations were also raised. In addition, cadmium appears to be in higher concentration in figs, peanuts and lemons as well as in grains and barley straw. Eating such materials cause a number of potential illnesses. Studies have also shown that heavy metal concentrations are high in native plant species such as *Dittrichia viscosa* (L.) Greuter and *Allium ampeloprasum*^75^. Iron-rich species have a range of both positive and negative effects on human health and this often depends on the structure of the iron compound concerned.

Historical records show that copper and copper compounds had been used medicinally at least as early as 400 BC^76^. Many copper compounds were used to treat a variety of diseases during the nineteenth century, and the presence of copper in plants and animals was recognized more than 150 years ago^77^. For quite some time it has been widely accepted that copper is an essential trace element required for survival by all organisms, from bacterial cells to humans^78^.

Waters draining from old mine areas also have the potential of having a range of dissolved minerals and salts^79^. In addition to primary dissolved iron and copper minerals there are many secondary minerals such as magnesium-, calcium-, sodium- and aluminium sulphate minerals and highly soluble iron sulphate salts. In both the ancient treatments^80^ as well as the modern we can use the structure of medical use groups proposed by Staub et al.^81^ that categorises treatments for a range of internal and external uses. This data can then be linked to known medical information concerning the importance of elements and hence be useful in helping to identify mineral species use in ancient pharmacological texts.

## Conclusion

Our project has so far established that the topic is far more complex than it would appear. Only very few plants are in the olive tree category, where we know conclusively what they were and whether they may have changed their properties over time. For the remainder, the names alone provide insufficient information due to their highly variable nature, over time and from place to place.

With inorganic materials, the balance is slightly different. We can be certain about a number of minerals, and in some cases we can narrow down what they would or would not have been. However, it still leaves us with a considerable number of items that are as such uncertain. What is more, once we move beyond basic lexicography, we realise how important minerals are in other ways, for instance as trace elements in water or absorbed in a plant. Moreover, minerals have to be considered in a much broader context than commonly thought.

In any case, the only way forward is to work in multidisciplinary teams, as this is a highly complex task.

## Figures and Tables

**Fig:**
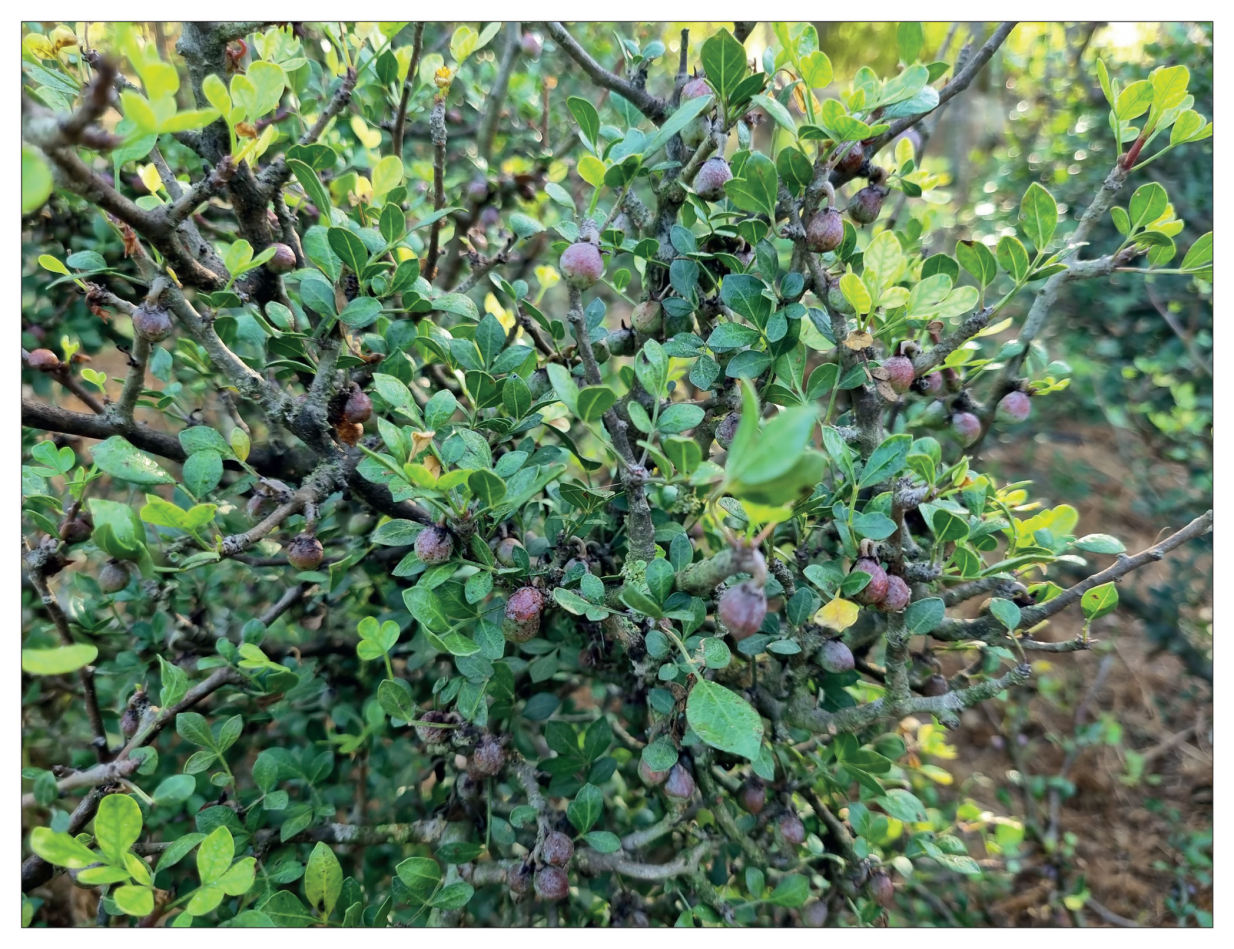
Picture of the plant in question. Photographed by P. Gan.

**Figure:**
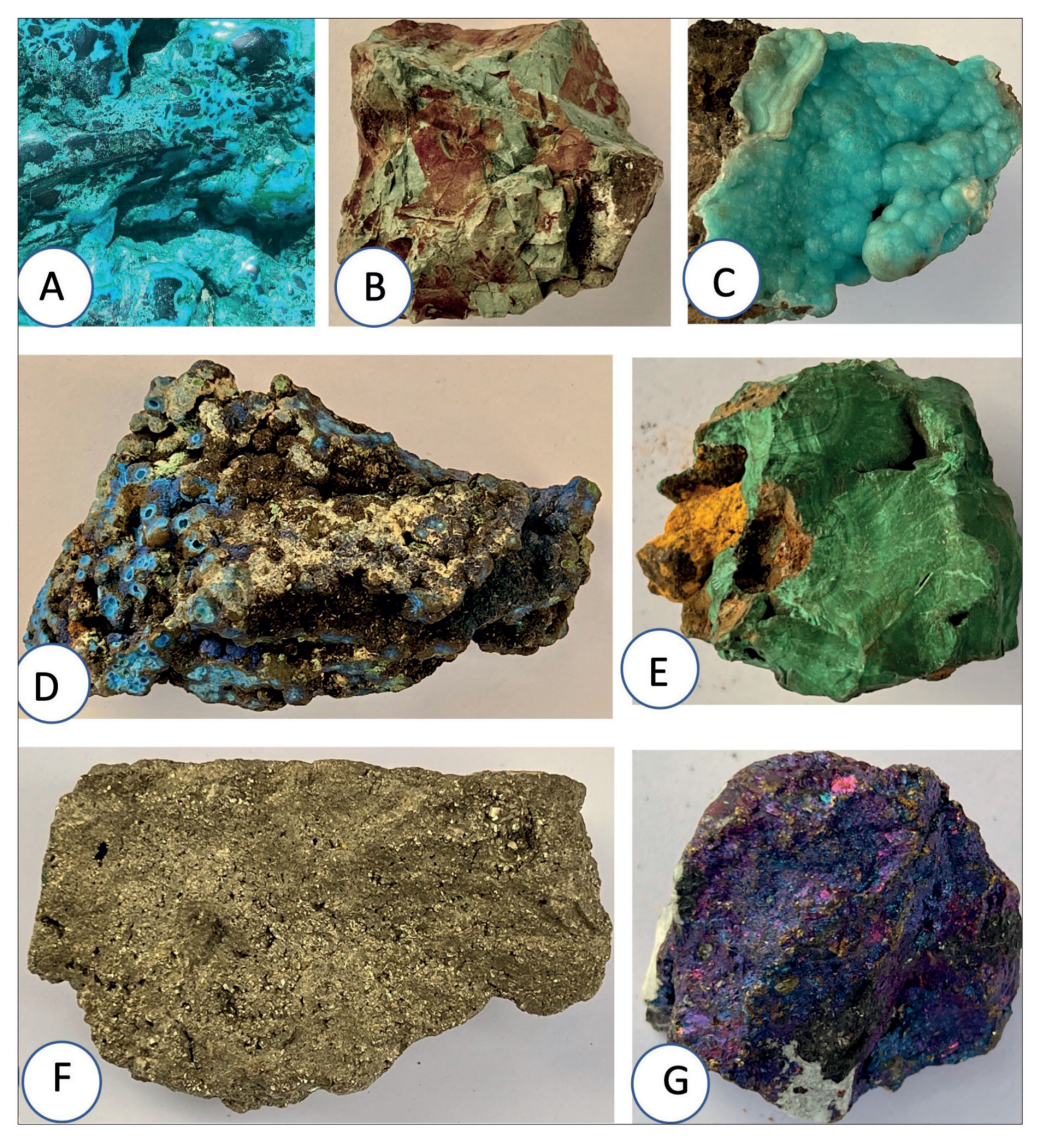
Plate. The geological samples A. Chrysocolla (Cu_2_H_2_Si_2_O_5_(OH)_4_), locality unknown. B. Celadonite (K(Mg,Fe2+)(Fe3+,Al)[Si4O10](OH)2). In pillow lavas, Klirou Bridge, Cyprus. C. Calamine (hemimorphite). Zinc silicate (Zn4(Si2O7)(OH)2·H2O), Cumberland, England D. Azurite (Cu3(CO3)2(OH)2), Bisbee, Arizona, U.S.A E. Malachite (Cu2CO3(OH)2.) Zambia, Africa. F. Copper ore comprising pyrite, chalcopyrite and covellite in propylitized lava. Limni Mine, Cyprus. G. Bornite Peacock ore. (Cu5FeS4), Cumberland, England.

## Footnotes

1 Our word ‘olive’ is related to the Greek word ἔλαιον, or rather its archaic form ἔλαιϝον, pronounced *elaivon*.

2 G. Besnard, B. Khadari, M. Navascués, M. Fernández-Mazuecos, A. El Bakkali, N. Arrigo, D. Baali-Cherif, V. Brunini-Bronzini de Caraffa, S. Santoni, P. Vargas, V. Savolainen, *The Complex History of the Olive Tree: From Late Quaternary Diversification of Mediterranean Lineages to Primary Domestication in the Northern Levant*, PRS.B 280(1756), 2013, p. 1–7. For further detail see C. Breton, M. Tersac, A. Bervillé, *Genetic Diversity and Gene Flow between the Wild Olive (Oleaster*, Olea europaea *L.) and the Olive: Several Plio*-*Pleistocene Refuge Zones in the Mediterranean Basin Suggested by Simple Sequence Repeats Analysis*, JBio 33, 2006, p. 1916–1928.

3 For recent discussion of ancient botanical writings with bibliography, see G. Hardy, L. Totelin, *Ancient Botany*, London–New York 2015. For further information see also A. Touwaide, *Pharmaceutical Literature*, [in:] *Handbook of Medieval Studies. Terms, Methods, Trends*, vol. I–III, ed. A. Classen, Berlin 2010, p. 1979–2000.

4 Medicinal Plant Names Services (2022), Royal Botanic Gardens, Kew, Version 11 [10 X 2022].

5 N.J. Turland, J.H. Wiersema, F.R. Barrie, W. Greuter, D.L. Hawksworth, P.S. Herendeen, S. Knapp, W.-H. Kusber, D.-Z. Li, K. Marhold, T.W. May, J. McNeill, A.M. Monro, J. Prado, M.J. Price, G.F. Smith, *International Code of Nomenclature for Algae, Fungi, and Plants (Shenzhen Code) Adopted by the Nineteenth International Botanical Congress Shenzhen, China, July 2017*, Glashütten 2018. For an in-depth discussion of the methodology see B. Allkin, K. Patmore, *Botanical Nomenclature for Herbal Medicines and Natural Products: its Significance for Pharmacovigilance*, [in:] *Pharmacovigilance for Herbal and Traditional Medicines*, ed. J. Barnes, Cham 2022.

6 In the present article, only a brief discussion will be presented. In depth information and a comprehensive picture of plant names mentioned in the bible can be found in A. Dafni, B. Böck, *Medicinal Plants of the Bible* – *Revisited*, JEE 15.1, 2019, p. 57. And further discussion can be found in D. Iluz, M. Hoffman, N. Gilboa-Garber, Z. Amar, *Medicinal Properties of Commiphora gileadensis*, AJPP 4, 2010, p. 516–520.

7 This story is transmitted in 1 Kings 10, 10. The Septuagint and the Vulgate use different titles to refer to this book of the bible.

8 Z. Amar, D. Iluz, *Balsam: the Most Expensive Perfume Plant in the Ancient World*, [in:] *The Paths of Daniel. Studies in Judaism and Jewish Culture in Honor of Rabbi Professor Daniel Sperber*, ed. A.S. Ferziger, Ramat Gan 2017, p. 15–27. This article offers an in depth analysis of the properties of *Commiphora gileadensis*. For further primary sources and discussion see also J.I. Miller, *The Spice Trade of the Roman Empire 29 B.C. to A.D. 641*, Oxford 1969.

9 *Biblia Hebraica Stuttgartensia*, ed. K. Ellinger, W. Rudolph, Stuttgart 1977.

10 *Hebräisches und aramäisches Handwörterbuch über das Alte Testament*, ed. W. Gesenius, F. Buhl, ^17^Berlin–Göttingen–Heidelberg 1962, *s.v*.

11 *Septuaginta id est Vetus Testamentum graece iuxta LXX interpretes*, ed. A. Rahlfs, Stuttgart 1979.

12 *LSJ, s.v*.

13 *Biblia Sacra iuxta Vulgatam Versionem*, ed. R. Weber, R. Gryson, Stuttgart 2007.

14 *Oxford Latin Dictionary*, ed. P.G.W. Glare, Oxford 2005, *s.v*.

15 *Flavii Iosephi opera*, ed. B. Niese, Berlin 1892 puts ὀποβαλσάμου in the edition and mentions the variant ἀποβαλσάμου in the apparatus, as transmitted by just one witness, and epitome of the work. The latest up to date edition, Flavius Josèphe, *Les Antiquités Juives*, vol. IV, ed. É. Nodet, Paris 2005, publishes ἀποβαλσάμου in the main text and does not list any variants in the apparatus.

16 *Λέξικο της μεσαιωνικης Ελληνικης δημώδους γραμματείας 1100*–*1669*, ed. E. Kriaras, Thessalonike 1968–2022, *s.v*. lists οποβάλσαμον as a lemma but not αποβάλςαμον. He describes it as the aromatic resin of the βαλσαμόδεντρον.

17 *Pedanii Dioscuridis Anazarbei de materia medica libri quinque*, vol. I–III, ed. M. Wellmann, Berolini 1907–1914.

18 See for instance a very recent example in A. Alhazmi, A.F. Aldairi, A. Alghamdi, A. Alomery, A. Mujalli, A.A. Obaid, W.F. Farrash, M. Allahyani, I. Halawani, A. Aljuaid, S.A. Alharbi, M. Almehmadi, M.S. Alharbi, A.A. Khan, M.A. Jastaniah, A. Alghamdi, *Antibacterial Effects of Commiphora gileadensis Methanolic Extract on Wound Healing*, Mole 27(10), 2022, p. 3320; L. Bouslama, B. Kouidhi, Y.M. Alqurashi, K. Chaieb, A. Papetti, *Virucidal Effect of Guggulsterone Isolated from Commiphora gileadensis*, PlM 85(16), 2019, p. 1225–1232; E. Amiel, R. Ofir, N. Dudai, E. Soloway, T. Rabinsky, S. Rachmilevitch, *β*-*Caryophyllene, a Compound Isolated from the Biblical Balm of Gilead (Commiphora gileadensis), Is a Selective Apoptosis Inducer for Tumor Cell Lines*, EBCAM 2012.

19 Due to the large volume of mentions throughout ancient and medieval literature, we can only present a selection of primary sources. For further data see for instance J. André, *Les noms de plantes dans la Rome antique*, Paris 1985.

20 *LSJ, s.v*.

21 *Pedanios Dioscorides of Anazarbus De Materia Medica*, II, 124, trans. L. Beck, Zürich–New York 2005, p. 144.

22 *Λέξικο της μεσαιωνικης*…, *s.v*. Kriaras also lists the variant spellings ἀτράχνη and ἀνδράγνη.

23 Cited from the online collection Loeb Classical Library, https://www-loebclassics-com.ezproxy01.rhul.ac.uk/view/theophrastus-enquiry_plants/1916/pb_LCL070.3.xml?rskey=DZKaYU&result=2 [29 IX 2022].

24 *Pline l’Ancient Historire Naturelle, livre XIII*, ed. et trad. A. Ernout, Paris 1956.

25 The transmission is not uniform, however, it is fair to assume that the above reconstructed text is correct. The latest edition cites the codex transmission of the first instance as *andraclen, andraclem* and *andraden*, and of the second instance *andrachne, andrache* and *andrahne*. Variant spellings of *ch* and *h* are common in Latin transmission, and it is easy to see how H and N could be confused in upper case letters. Moreover, *cl* and *d* could easily be mistaken for each other. *Pline l*’*Ancient Historire Naturelle, livre XIII*, ed. et trans. A. Ernout, Paris 1956. An earlier edition comes to similar readings, adding also the variants *andrachie* and *andrachye* in a pinax. *C. Plinii Secundi Naturalis Historiae libri XXXVII*, vol. I, ed. I. Sillig, Hamburgi–Gothae 1851.

26 One of these would commonly be identified with two species of the strawberry tree, *Arbutus unedo* L. and *Arbutus andrachne* L., a characteristic element of the Mediterranean sclerophyllous forest, the other with the herb *Portulaca oleracea* L.

27 *Pedanii Dioscuridis Anazarbei*…

28 *Anecdota Atheniensia et alia*, vol. II, ed. A. Delatte, Paris 1939, p. 385–393. P. 386, 22f.

29 *Anecdota Atheniensia*…, p. 373.13.

30 Oribasius, *Collectiones Medicae*, XII π 9, 3. *Oribasii Collectionum Medicarum Reliquiae*, vol. II, ed. I. Raeder, Lipsiae–Berolini 1929.

31 *Corpus hippiatricorum Graecorum*, vol. I, ed. E. Oder, C. Hoppe, Leipzig 1924 [repr. 1971]. This edition lists no variant readings in this passage.

32 These entries are cited from simonofgenoa.org *s.v. portulaca*, http://simonofgenoa.org/index.php?title=Portulaca [29 IX 2022] and *s.v. andragne*, http://simonofgenoa.org/index.php?title=Andragne [29 IX 2022] and we acknowledge the work of Wilf Gunther on these lemmata.

33 *Arabisches Woerterbuch fuer die Schriftsprache der Gegenwart*, ed. H. Wehr, ^5^Wiesbaden 1985, *s.v*. BQL.

34 E. Lev, Z. Amar, *Practical Materia Medica of the Medieval Eastern Mediterranean According to the Cairo Genizah*, Leiden 2008, p. 253–255 provides extensive discussions of the corresponding medieval Hebrew term and the medicinal application described in all sources. See also this article for further secondary literature.

35 *John the Physician*’*s Therapeutics. A Medical Handbook in Vernacular Greek*, ed. B. Zipser, Leiden 2009.

36 See for instance Vitruvius’ first century BC account of vermillion and mercury, as described in VII 8. The description of the mineral and its physical properties allow for a conclusive identification. *Vitruvii de architectura libri decem*, ed. V. Rose, Lipsiae 1899.

37 W. Schumann, *Gemstones of the World*, London 1977, p. 100. The name sapphire used to be applied to various stones. In antiquity and as late as the middle ages the name sapphire was understood to mean what is today described as lapis lazuli.

38 J. Bauer, *A Field Guide in Colour to Minerals, Rocks and Precious Stones*, London 1974.

39 W.A. Deer, R.A. Howie, J. Zussmann, *An Introduction to the Rock*-*forming Minerals*, ^3^London 2013.

40 *Ibidem*; J. Bauer, *A Field Guide*…

41 R.M. Hazen, S.M. Morrison, *On the Paragenetic Modes of Minerals: A Mineral Evolution Perspective*, AMin 107, 2022, p. 1262–1287; R.M. Hazen, S.M. Morrison, S.V. Krivovichev, R.T. Downs, *Lumping and Splitting: Toward a Classification of Mineral Natural Kinds*, AMin 107, 2022, p. 1288–1301.

42 A. Lu, *Mineral Evolution Heralds a New Era for Mineralogy*, AMin 107, 2022, p. 1217–1218.

43 As above, we used both Wellmann’s edition and Beck’s translation as a basis. See also T.A. Osbaldeston, R.P. Wood, *Dioscorides De Materia Medica. Being an Herbal with Many Other Medicinal Materials Written in Greek in the First Century of the Common Era*, Johannesburg 2000.

44 *Theophrastus On Stones*, ed. et trans. E. Caley, J. Richards, Columbus Ohio 1956, p. 39, 40.

45 A.-J.F.-M. Brochant de Villiers, *Traité élémentaire de mineralogique*, vol. I–II, ^2^Paris 1808.

46 J. Bauer, *A Field Guide*…, p. 102.

47 W.A. Deer, R.A. Howie, J. Zussmann, *An Introduction*…, p. 254.

48 V.A. Dritss, B.B. Zviagina, D.K. McCarty, A.L. Salyn, *Factors Responsible for Crystal*-*chemical Variations in the Solid Solutions from Illite to Aluminoceladonite from Glauconite to Celadonite, Sample 69*, AMin 95, 2010, p. 348–361.

49 M.R. Cleintuar, G.J. Knox, P.J. Ealey, *The Geology of Cyprus and its Place in the East*-*Mediterranean Framework*, GMij 56, 1977, p. 66–82; G. Constantinou, J.S. Govett, *Geology, Geochemistry, and Genesis of Cyprus Sulfide Deposits*, EGeo 68, 1973, p. 843–858; O. Davies, *The Copper Mines of Cyprus*, ABSA 30, 1928/1929 – 1929/1930, p. 74–85.

50 N. Antivachis, *The Geology of the Northern Part of the Apliki Cyprus*-*type Ore Deposit*, BGSG 49, 2015, p. 4–28; D.N. Antivachis, E. Elias Chatzitheodoridis, N. Skarpelis, K. Komnitsas, *Secondary Sulphate Minerals in a Cyprus*-*Type Ore Deposit, Apliki, Cyprus: Mineralogy and Its Implications Regarding the Chemistry of Pit Lake Waters*, MWE 36, 2017, p. 226–238; L. van Brempt, V. Kassianidou, *Facing the Complexity of Copper*-*sulphide Ore Smelting and Assessing the Role of Copper in South*-*central Cyprus: A Comparative Study of the Slag Assemblage from Late Bronze Age Kalavasos*-*Ayios Dhimitrios*, JAS.R 7, 2016, p. 539–553.

51 P. 273.

52 W.A. Deer, R.A. Howie, J. Zussmann, *An Introduction*…, p. 376.

53 W.A. Deer, R.A. Howie, J. Zussmann, *An Introduction*…; J. Bauer, *A Field Guide*…

54 R.B. Finkelman, H.C.W. Skinner, G.S. Plumlee, J.E. Bunnell, *Medical Geology*, Geot 46, 2001, p. 20–23; *Essentials of Medical Geology*, ed. O. Selinus, B. Alloway, J.A. Centeno, R.B. Finkelman, R. Fuge, U. Lindh, P. Smedley, New York 2005.

55 *Essentials of Medical Geology*…

56 M.C. Linder, M. Hazegh-Azam, *Copper Bio*-*chemistry and Molecular Biology*, AJCN 63, 1996, p. 797S–811S.

57 W.G. Ernst, *Earth Materials and Human Health*, IGR 48, 2006, p. 191–208; *Essentials of Medical Geology*…

58 H.H.A. Dolwett, J.R.J. Sorenson, *Historic Uses of Copper Compounds in Medicine*, TEM 2.2, 1985, p. 80–87.

59 J. Bauer, *A Field Guide*…

60 O. Davies, *The Copper Mines*…

61 *Ibidem*; *LSJ, s.v*. κύπριος.

62 A.B. Knapp, V. Kassianidou, M. Donnelly, *Copper Smelting in Late Bronze Age Cyprus: The Excavations at Politiko Phorades*, NEA 64.4, 2001, p. 204–210. Particularly useful in this context is T. Greensmith, *Southern Cyprus. Geologist*’*s Association Guide No 50*, London 1994.

63 S. Edwards et al., *Classic Geology in Europe. Cyprus*, Edinburgh 2010; T. Greensmith, *Southern Cyprus*…

64 O. Davies, *The Copper Mines*…; N. Antivachis, *The Geology*…; D.N. Antivachis, E. Elias Chatzitheodoridis, N. Skarpelis, K. Komnitsas, *Secondary Sulphate Minerals*…

65 D.G. Eliopoulos, M. Economou-Eliopoulos, G. Economou, V. Skounakis, *Mineralogical and Geochemical Constraints on the Origin of Mafic*–*Ultramafic*-*Hosted Sulphides: The Pindos Ophiolite Complex*, Min 10, 2020, p. 454.

66 L. van Brempt, V. Kassianidou, *Facing the Complexity*…

67 O. Davies, *The Copper Mines of Cyprus*…; N. Antivachis, *The Geology*…; D.N. Antivachis, E. Elias Chatzitheodoridis, N. Skarpelis, K. Komnitsas, *Secondary Sulphate Minerals*…

68 D.G. Eliopoulos, M. Economou-Eliopoulos, G. Economou, V. Skounakis, *Mineralogical and Geochemical Constraints*…

69 *Essentials of Medical Geology*…

70 *Essentials of Medical Geology*…

71 G. Borkow, J. Gabbay, *Copper, An Ancient Remedy Returning to Fight Microbial, Fungal and Viral Infections*, CChB 3, 2009, p. 272–278.

72 S.I. Hajdu, *Pathfinders in Oncology from Ancient Times to the End of the Middle Ages*, Can 122, 2016, p. 1638–1646.

73 L. van Brempt, V. Kassianidou, *Facing the Complexity*…

74 A. Christou, P. Christodoulos, C.P. Theologides, C. Costa, K. Ioannis, I.K. Kalavrouziotis, P. Soterios, S.P. Varnavas, *Assessment of Toxic Heavy Metals Concentrations in Soils and Wild and Cultivated Plant Species in Limni Abandoned Copper Mining Site, Cyprus*, JGEx 178, 2017, p. 16–22.

75 *Ibidem*.

76 K.E. Mason, *A Conspectus of Research on Copper Metabolism and Requirements of Man*, JNu 109, 1979, p. 1079–2066.

77 W.G. Ernst, *Earth Materials*…

78 M.C. Linder, M. Hazegh-Azam, *Copper Bio*-*chemistry*…

79 D.N. Antivachis, E. Elias Chatzitheodoridis, N. Skarpelis, K. Komnitsas, *Secondary Sulphate Minerals*…

80 E. Lev, *Healing with Minerals and Inorganic Substances: A Review of Levantine Practice from the Middle Ages to the Present*, IGR 52.7–8, 2010, p. 700–725; U.G. Limpitlaw, *Ingestion of Earth Materials for Health by Humans and Animals*, IGR 52.7–8, 2010, p. 726–744.

81 P.O. Staub, M.S. Geck, C.S. Weckerle, L. Casu, M. Leonti, *Classifying Diseases and Remedies in Ethnomedicine and Ethnopharmacology*, JEph 174, 2015, p. 514–519.
